# Comparing two frailty concepts among older people with intellectual disabilities

**DOI:** 10.1007/s10433-016-0388-x

**Published:** 2016-06-24

**Authors:** Josje D. Schoufour, Michael A. Echteld, Heleen M. Evenhuis

**Affiliations:** 1 Intellectual Disability Medicine, Department of General Practice, Erasmus Medical Center, PO box 2040, 3000 CA Rotterdam, The Netherlands; 2 Department of Epidemiology, Erasmus Medical Center, PO box 2040, 3000 CA Rotterdam, The Netherlands

**Keywords:** Frailty, Frailty index, Frailty phenotype, Survival, Intellectual disability

## Abstract

In general, disabilities are considered a consequence of frailty rather than a cause of frailty, whereas in people with intellectual disabilities (ID), disabilities are often lifelong, which could have consequences for the feasibility and validity of frailty instruments. To better understand frailty in people with ID, we compared two broadly used concepts: the frailty phenotype (FP) and the frailty index (FI) taking into account their feasibility (e.g., percentage of participants able to complete the frailty assessments), agreement, validity (based on 5-year mortality risk), influence of motor disability, and the relation between single frailty variables and mortality. The FI and an adapted version of the FP were applied to a representative dataset of 1050 people with ID, aged 50 years and over. The FI was feasible in a larger part of the dataset (94 %) than the adapted FP: 29 % for all five items, and 81 % for at least three items. There was a slight agreement between the approaches (*κ* = 0.3). However defined, frailty was related with mortality, but the FI showed higher discriminative ability and a stronger relation with mortality, especially when adjusted for motor disabilities. Concluding, these results imply that the used FI is a stronger predictor for mortality and has higher feasibility than our adaptation of the FP, in older people with ID. Possible explanations of our findings are that we did not use the exact FP variables or that the FI includes multiple health domains, and the variables of the FI have lower sensitivity to lifelong disabilities and are less determined by mobility.

## Introduction

Frailty is a complex cascade that involves several age-related physiological alterations, eventually leading to loss of function and failure to respond to a stressor event (Clegg et al. 2013). The frailty ‘phenotype’ by Fried et al. (2001), and the frailty index (FI) developed by Rockwood and Mitnitski (Mitnitski et al. 2001; Rockwood and Mitnitski 2007) are the most evaluated and most frequently used measures, representing two different concepts and definitions of frailty (Bouillon et al. 2013).

The frailty phenotype was operationalized as a biological syndrome, based on a cluster of symptoms that are commonly observed in frail older people including unintended weight loss, low grip strength, exhaustion, slow gait speed, and low physical activity (Fried et al. 2001). The underlying concept is the cycle of frailty, based on age-related physiological changes, including low energy expenditure, nutritional deficiencies, and sarcopenia. Because the frailty phenotype has a clear underlying etiology, it is, although correlated to, distinct from disabilities and chronic disease (Fried et al. 2004). The frailty phenotype distinguishes between three different frailty states, based on the number of symptoms present in a person. If none of the symptoms are present, a person is classified as non-frail, one or two marks a person as pre-frail, and frailty is defined as the presence of three or more. The frailty phenotype can relatively easily be used as a clinical assessment for frailty, using clearly described cut-off values.

The FI operationalizes frailty quantitatively as an aspecific accumulation of health deficits in multiple domains, and can include health variables such as signs, diseases, disabilities, laboratory abnormalities, and symptoms as long as they are not too common or too rare, generally increase with age, and together cover several health domains. In order to find an FI that best captures the risk for adverse health outcomes, it has been suggested to also include disabilities and diseases (Theou et al. 2012). The FI-score is a continuous value between 0 and 1. Because of the continuous nature of the FI, it is possible to study individual changes over time, as frailty is usually an irregular trajectory influenced by stress and recovery (Mitnitski et al. 2012). On the other hand, on a population level, the FI shows very regular characteristics including an exponential relation with age (Mitnitski et al. 2013).

The two approaches agree that, as a consequence of multisystem deterioration, frailty is an age-related state of vulnerability to adverse health outcomes (Theou and Rockwood 2015). Nevertheless, because of different underlying conceptualizations and operationalizations, it has been suggested not to compare the two instruments but rather consider them as complementary to one other (Cesari et al. 2014). Even so, to better understand frailty in populations with different characteristics, it can be helpful to compare different concepts. Previous comparisons showed that the FI has a somewhat stronger relation with negative health outcomes than the phenotype approach (Theou and Rockwood 2015).

In people with intellectual disabilities (ID), insights into how frailty originates, develops, and affects health outcomes are currently speculative. Results from the general population cannot directly be applied to this group, because of their lifelong disabilities (cognitive, motor, and sensory) and chronic comorbidity, which may influence both the development and the consequences of frailty. Moreover, muscle function and mobility play a central role in the frailty phenotype, whereas these frailty characteristics could be lifelong in people with ID and highly determine the frailty status (Evenhuis et al. 2013).

A better understanding of the different concepts of frailty in people with ID can help improve the understanding of frailty in people with ID and better understand how people with ID age. Furthermore, it provides insight and direction for a future-screening instrument for frailty in this lifelong disabled population.

The main aim of our study was therefore to compare the feasibility and validity of the frailty phenotype and the frailty index in older adults with ID. First, feasibility was assessed by evaluating the percentage of participants able to complete the frailty assessments. Second, validity was assessed. Because there is no gold standard for frailty, validity is usually based on criterion validity, in this case the relation with adverse health outcomes. We therefore calculated the relation between frailty and 5-year survival. Third, in addition to general feasibility and criterion validity, we were interested in the predictive value of the single items that are part of the frailty phenotype and the FI in order to find risk factors that highly contribute to the mortality risk of people with ID. Last, because lifelong motor disabilities can highly determine the frailty status, we evaluated the influence of motor disability on the frailty status.

## Methods

### Study design and participants

This study was part of the HA-ID study. This study addressed the health of 1050 older people with ID in the Netherlands. Details about the recruitment and selection process have been described elsewhere (Hilgenkamp et al. 2011). Briefly, the study sample consisted of clients, aged 50 years and over, from three Dutch care provider services offering a broad spectrum of care and support to people with ID. All clients aged 50 years and over (*N* = 2322) were invited to participate. Eventually 1050 clients, or their legal representatives, provided informed consent, forming a nearly representative study sample for the Dutch population of older adults (aged 50 and above) with ID who use formal care, albeit with a slight underrepresentation of men, people aged 80 and over, and people living independently. Ethical clearance was provided by the Medical Ethics Committee of the Erasmus Medical Center Rotterdam (MEC 2008-234) and by the ethics committees of the participating care organizations. The study followed the guidelines of the Declaration of Helsinki.

### Data collection

Baseline data were collected between February 2009 and July 2010 within three main themes: (1) physical activity and fitness, (2) nutrition and nutritional state, and (3) mood and anxiety. Within these themes the participants underwent an extensive diagnostic assessment including a physical assessment, a fitness test battery, several questionnaires (regarding, e.g., nutrition, depression, disabilities), and laboratory tests in addition to the collection of health record data. Data on age, gender, and residential status were collected through the care provider services. Level of ID was obtained from the scores determined by psychologists or test assistants from available IQ tests. The diagnosis of Down syndrome was retrieved from medical files. Up to March 2015, all-cause mortality data (time of death) were collected through the care organizations.

### Frailty measures

#### Frailty phenotype

Previously, an adapted version of the frailty phenotype was applied using the criteria of the Cardiovascular Health Study (Evenhuis et al. 2012; Fried et al. 2001). According to the original criteria, an individual should be classified as frail if at least three of the following five are present: weight loss, weakness, slowness, low physical activity, and poor endurance or exhaustion. Briefly, weight loss was defined as losing more than 3 kg within 3 months. Weakness was assessed using a handgrip dynamometer. Slowness was assessed using comfortable walking speed, measured as the average of three recordings of the time to complete a distance of 5 m. Participants in a wheelchair and participants unable to perform the walking test due to physical limitations were also classified as having a slow walking speed. Low physical activity was defined as walking fewer than 5000 steps/day measured with pedometers (NL-1000; New Lifestyles, Lees Summit, MO). Participants in a wheelchair and participants unable to perform the test due to physical limitations were also classified as having low physical activity (less than 5000 steps/day). Exhaustion was defined as answering ‘moderate problem’ or ‘severe problem’ to the ‘lacks energy’ item from the Anxiety, Depression, and Mood Scale (Esbensen et al. 2003). Additional information about the used frailty phenotype variables and the originally intended frailty phenotype criteria are provided in Table 5. Individuals with one or two criteria present were classified as pre-frail. Individuals with no criteria present were classified as non-frail or ‘robust.’ At least three out of five criteria needed to be known before the frailty phenotype could be applied.

#### Frailty index

An FI was previously created with 51 baseline items from the HA-ID study (Schoufour et al. 2013). A standardized procedure was followed to develop the FI (Searle et al. 2008): all items were (1) related to health, (2) positively associated with age, (3) frequently but not too often present in the population (>5 %, <80 %), and (4) measured in at least 70 % of the participants. Furthermore, the items did not correlate too strongly with each other (*r* < 0.7), and together the items covered a range of health problems (physical, psychological, and social). Deficits included are, for example, mobility, calf circumferences, bathing, falling, listless, grip strength, HDL cholesterol, and knowing which year it is. An overview of all the deficits included in the FI is provided in Table 4. All items were recorded between 1 (presence of the deficit) and 0 (absence of the deficit). The FI-score was calculated as the total number of deficits present as a proportion of those counted (e.g., 12 deficits in a 51-item FI results in an FI of 12/51 = 0.24). In the case of missing data, the deficit was removed from both the numerator and the denominator, but at least 30 deficits were required per individual. For the sake of direct comparisons with Fried phenotype, we used several previously identified cut points. Because using cut points for the FI is generally not advised and cut-offs are arbitrary, we applied three different, previously used, cut-offs for the FI. First of all, an FI of less than 0.2 was considered as non-frail or ‘robust,’ a score between 0.2 and 0.35 as ‘pre-frail,’ and a score above 0.35 as ‘frail’ (Kulminski et al. 2008; Rockwood et al. 2007). Second, an FI-score of ≤0.08 was considered non-frail, a score between 0.08 and 0.25 as pre-frail and a score equal to or higher than 0.25 as frail, in accord with prior studies (Rockwood et al. 2007; Rockwood et al. 2004; Song et al. 2010). Third, we classified participants as non-frail if FI ≤0.10, frail if FI ≥0.21, and pre-frail if the score was between 0.10 and 0.21, identified using stratum specific likelihood ratios (Hoover et al. 2013).

### Statistical analysis

First, baseline characteristics (gender, age, level of ID, presence of Down syndrome), the mean FI-score and the percentage of non-frail, pre-frail, and frail participants were provided as the percentage for categorical variables and the mean (with *SD*) for continuous variables. The prevalence of each item of the phenotype was provided. Second, the feasibility of the instruments was analyzed by calculating the percentage of participants able to complete the frailty assessments. A non-response analysis was performed to compare the participants with and without completed data for frailty, using a Pearson Chi-square for categorical data and ANOVA for continuous data. Third, the Cohen’s Kappa statistic was used to estimate agreement between the instruments. For this analysis, the categorized FI was compared with the frailty phenotype (e.g., non-frail, pre-frail, frail). Agreement was considered as poor for Kappa values lower than 0.21, slight for 0.21–0.40, moderate for 0.41–0.60, good for 0.61–0.80, and excellent for values 0.81–1 (Cohen 1960). Fourth, the ability to predict 5-year all-cause mortality was calculated for both instruments and compared to each other. The hazard ratio’s (*HR*) for mortality were calculated for the frailty phenotype and for the categorized FI in separate Cox regression models. Dummy variables were composed for the pre-frail and frail groups to compare their mortality risk with the non-frail group. Additionally, the *p* for trend was provided. A comparative analysis was performed by including the two frailty instruments in one Cox regression model. This analysis was repeated with an FI that excluded the criteria that were also used for the frailty phenotype. In other words, the deficits' walking speed, grip strength, fatigue, and weight loss were excluded from the FI. All models were adjusted for age (years), level of ID (with dummy variables for moderate and severe/profound), gender and the presence of Down syndrome. A receiver operating characteristics (ROC) curve was constructed and the area under the ROC curve (AUC) was calculated to measure the discriminative ability of the instruments in relation to survival. These calculations were based on the Nearest Neighbor Estimator, which uses time-dependent ROC and AUC, to account for censoring (Heagerty et al. 2000). In order to find the frailty variables that explained most variance in survival time, we calculated the *HR* for each FI item and for each frailty phenotype item. Additionally, we added all available frailty measures (frailty phenotype and FI) into a forward Cox Regression model. Full case analysis resulted in a small and very selective group. Therefore, for this analysis, we used a multiple imputation procedure using fully conditional specification (Markov chain Monte Carlo method) with a maximum of 100 iterations. In total, we created 10 imputed datasets using all the frailty measures as predictors in addition to the baseline characteristics—Down syndrome, age, gender, and level of ID. Because we used a stepwise entry of the variables, leading to different predictor sets for the various imputation sets, pooling of the results was impossible. We therefore provide the results of the 10th imputation set. Fifth, the influence of motor disability was assessed by including motor disability into a Cox regression model.

For all survival analyses, the data on participants who were lost to follow-up were censored and the proportional Hazards assumption was tested with the scaled Schoenfeld residuals. Statistical analyses were performed using SPSS version 20.0 and R version 3.0.0. A two-sided *p* value of <0.05 was considered significant.

## Results

### Sample characteristics

The mean age of the study sample (*n* = 1050) was 61.6 (SD = 8.0). Nearly half were female (*n* = 511, 49 %), nearly half had a moderate level of ID (*n* = 506, 48 %), and 14 % (*n* = 149) was diagnosed with Down syndrome. According to the frailty phenotype, 230 (27 %) were classified as non-frail, 508 (60 %) as pre-frail, and 110 (13 %) as frail. The mean FI-score was 0.27 (SD = 0.13). Using the first defined cut-off (non-frail < 0.2, frail >0.35), 325 (33.1 %) participants were classified as non-frail, 392 as pre-frail (37.3 %), and 265 as frail (25.2 %) according to the FI. According to the second used cut-off (non-frail ≤0.08, frail ≥0.25), 33 (3.4 %) were non-frail, 445 (45 %) pre-frail, and 504 (51 %) frail. The third applied cut-off (non-frail ≤0.10, frail ≥0.21) classified 65 (6.6 %) participants as non-frail, 285 (29 %) as pre-frail, and 632 (64 %) as frail.

### Feasibility

Less than a third of the participants (*n* = 307, 29 %) could complete the full frailty phenotype assessment as intended. 40 % (*n* = 419) had four completed assessments, 12 % (*n* = 122) had three completed assessments, and 19 % (*n* = 202) had less than three completed assessments. By including all participants with at least three known frailty phenotype criteria, the frailty phenotype could be applied to 848 (81 %) participants. Table 1 provides an overview of the feasibility of the single frailty phenotype variables. The 202 excluded participants were on average more intellectually disabled (*X* ^2^ = 32.8, *p* < 0.001), and had on average a higher FI-score (*M* = 0.31, SD = 0.12) than those included ([*M* = 0.27, SD = 0.13], *t*(982) = 3.28, *p* = 0.001). For other baseline characteristics, no significant differences between the included and excluded participants were found. For 167 participants (17.2 %), all 51 included deficits were known. In 68 (6.4 %) participants, there was too much missing data to calculate an FI. There were no significant associations between the number of missing data and the FI-score or between the participants with a known FI-score (*n* = 982, 94 %) and those without, with respect to gender, age, level of ID, and Down syndrome.

**Table 1:** The variables of the frailty phenotype, feasibility, and association with survival and the frailty index

Association with	Feasible (%)	Classified as frail for this item n (%)	Mortality HR (95 % CI)^a^	Frailty index *B* (SE)^a^
Grip strength	977 (93)	46 (4.4)	2.06 (1.13–3.74)	0.07 (0.06–0.09)
Weight loss	725 (69)	384 (53)	1.93 (1.13–3.31)	0.08 (0.05–0.11)
Exhaustion	975 (92)	171 (18)	1.95 (1.36–2.81)	0.11 (0.09–0.13)
Slow walking speed	818 (78)	271 (33)	3.64 (2.31–5.75)	0.15 (0.14–0.17)
Physical inactivity	422 (10)	255 (60)	5.43 (2.07–14.3)	0.14 (0.11–0.16)

^a^HR and B are adjusted for age, gender, level of ID, and the presence of Down syndrome. The regression coefficient B represents differences in absolute frailty index score (and corresponding 95 % confidence interval)

### Agreement

For 838 participants, the frailty phenotype and the FI were known. The Cohen’s Kappa agreement between the three categorized FIs and the frailty phenotype ranged between 0.10 and 0.30, corresponding with poor till fair agreement (Table 2). From the three applied FI cut-off values, only the first (non-frail <0.2; frail >0.35) showed a fair agreement with the frailty phenotype (Kappa agreement 0.3). The two other applied FI cut-off points showed poor agreement with the frailty phenotype (Kappa agreement 0.10 and 0.11). Each frailty phenotype variable was independently of age, level of ID, gender, and Down syndrome significantly associated with the FI (Table 1).

**Table 2:** Agreement among the frailty index (using different cut-off values) and the frailty phenotype based on three frailty categories

*n* = 838^a^		Frailty phenotype				
		Non-frail	Pre-frail	Frail	Total	Agreement
Frailty index	Non-frail <0.2	151	146	2	299	0.30
	Pre-frail 0.2–0.35	68	232	27	327	
	Frail >0.35	5	126	81	212	
	Total	224	504	110	838	
Frailty index	Non-frail ≤0.08	23	5	0	28	0.10
	Pre-frail 0.08–0.25	162	235	8	405	
	Frail ≥0.25	39	264	102	405	
	Total	224	504	110	838	
Frailty index	Non-frail ≤0.10	45	12	0	57	0.11
	Pre-frail 0.10–0.21	110	151	2	263	
	Frail ≥0.21	69	341	108	518	
	Total	224	504	110	838	

^a^From the total HA-ID population (*n* = 1050), 838 had a known frailty phenotype and a frailty index score

### Survival

Of the total HA-ID cohort (*n* = 1050), 207 participants died during the follow-up. Table 3 shows the HR’s for pre-frail and frail individuals, using the non-frail state as a reference group, and the p for trend across all categories. However defined, frailty was significantly related to mortality. Those classified as pre-frail or frail using the frailty phenotype were, respectively, 2.04 and 4.20 times more likely to die during the follow-up period than those classified as non-frail. Those classified as pre-frail or frail with the FI (using the <0.2 to define robust, and >0.35 to define frail participants) were, respectively, 2.27 and 10.3 times more likely to die than the non-frail group. If both instruments were included in one Cox regression model, the frailty phenotype no longer predicted mortality, whereas the FI did. If all frailty phenotype items were excluded from the FI, virtually the same results were obtained. Although the HR for the frailty phenotype groups slightly increased, they remained not significant (data not shown). For the two FI scores that used the lowest cut-off of 0.08 or 0.10, two participants died in the reference group. There was no association with mortality in the pre-frail and frail participants compared to the reference group. The p for trend was highly significant. Repeating the analysis with all participants with a known FI (*n* = 982) revealed somewhat stronger associations between the FI and mortality (Table 6). The ROC curve showed that the first categorized FI (robust <0.25, frail >0.35) had a higher discriminative ability in relation to all-cause mortality (AUC = 0.78) than the frailty phenotype (AUC = 0.64). The second (robust ≤0.08, frail ≥0.25) and third cut values (robust ≤0.10, frail ≥0.21) had an AUC of 0.69 and 0.66, respectively.

**Table 3:** Hazard ratio’s for 5-year all-cause mortality according to the three level frailty index, using three sets of cut-off values, and the frailty phenotype

Frailty measure	Status	*N* = 818 † = 164		Single frailty instrument			Both frailty instruments			Motor disability		
		n cat	† cat	HR (95 % CI)	Wald	*p*	HR (95 % CI)	Wald	*p*	HR (95 % CI)	Wald	*p*
Frailty phenotype	Non-frail	221	19	Reference		Trend: <0.001	Reference		Trend: 0.90	Reference		Trend: 0.03
	Pre-frail	488	100	2.04 (1.23–3.37)	7.74	0.005	1.12 (0.65–1.92)	0.17	0.68	1.70 (1.01–2.84)	4.05	0.04
	Frail	109	45	4.20 (2.39–7.39)	24.9	<0.001	1.16 (0.62–2.19)	0.22	0.64	2.31 (1.24–4.32)	6.96	0.008
Frailty index	<0.2	295	19	Reference		Trend: <0.001	Reference		<0.001	Reference		<0.001
	0.2–0.35	317	44	2.27 (1.31–3.93)	8.52	0.005	2.19 (1.24–3.87)	7.20	0.007	2.22 (1.28–3.85)	7.96	0.005
	>0.35	206	101	10.3 (5.97–17.9)	69.6	<0.001	9.66 (5.23–17.8)	52.5	<0.001	8.53 (4.69–15.5)	49.4	<0.001
Frailty phenotype	Non-frail	221	19	Reference		Trend: <0.001	Reference		Trend: 0.019	Reference		Trend: 0.03
	Pre-frail	488	100	2.04 (1.23–3.37)	7.74	0.005	1.42 (0.84–2.38)	1.71	0.008	1.70 (1.01–2.84)	4.05	0.04
	Frail	109	45	4.20 (2.39–7.39)	24.9	<0.001	2.21 (1.23–4.00)	6.93	0.02	2.31 (1.24–4.32)	6.96	0.008
Frailty index	≤0.08	28	2	Reference		Trend: <0.001	Reference		Trend: <0.001	Reference		Trend: <0.001
	0.08–0.25	398	32	0.94 (0.22–3.99)	0.01	0.94	0.84 (0.20–3.58)	0.06	0.81	0.96 (0.23–4.08)	0.00	0.96
	≥0.25	392	130	3.98 (0.95–16.7)	3.55	0.06	2.93 (0.67–12.7)	2.06	0.15	3.15 (0.74–13.4)	2.42	0.12
Frailty phenotype	Non-frail	221	19	Reference		Trend: <0.001	Reference		Trend: 0.001	Reference		Trend: 0.03
	Pre-frail	488	100	2.04 (1.23–3.37)	7.74	0.005	1.46 (0.87–2.43)	2.05	0.54	1.70 (1.01–2.84)	4.05	0.04
	Frail	109	45	4.20 (2.39–7.39)	24.9	<0.001	2.56 (1.44–4.56)	10.2	0.03	2.31 (1.24–4.32)	6.96	0.008
Frailty index	≤0.10	56	2	Reference		Trend: <0.001	Reference		Trend: <0.001	Reference		Trend: <0.001
	0.10–0.21	260	19	1.79 (0.41–7.75)	0.61	0.44	1.59 (0.36–6.96)	0.39	0.15	1.86 (0.43–8.05)	0.69	0.41
	≥0.21	502	143	6.55 (1.58–27.2)	6.72	0.01	4.79 (1.12–20.4)	4.48	0.001	5.31 (1.27–22.2)	5.25	0.02

*Note* *HR* hazard ratio, *CI* confidence interval, *†* the total number of deceased participants, the non-frail state was used a reference category for each frailty instrument. All models were adjusted for age, gender, level of ID, and Down syndrome. Participants were excluded if they had missing data on one of the frailty instruments, motor disabilities, or other covariates (*n* = 232). The model *motor disability* was adjusted for the level of motor impairment, ‘no walking impairment’ was used as a reference category; the model for motor disability included only the frailty phenotype or the frailty index

Almost all single frailty items were associated with survival (Tables 1, 4). Mobility-related items of the frailty phenotype (e.g., walking speed and physical activity) were more strongly associated with both mortality and the FI than the other items (e.g., grip strength, weight loss, and exhaustion). The forward regression analysis showed that a broad range of variables, including walking stairs, present at the day care center, panic attacks, asthma/COPD and hemoglobin, and fast fatigue, were selected as independent predictors for survival (Table 7).

### Motor disability and frailty

Information on mobility was known for 989 participants. At baseline, 731 (74 %) participants walked independently, 151 (15 %) walked with support, and 107 (11 %) were wheelchair dependent. Those who walked with support were 2.03 (95 % CI = 1.40–2.97) times, and those who were wheelchair dependent were 4.10 (95 % CI = 2.83–5.96) times, more likely to have deceased during the follow-up compared to those who walked independently. The last column in Table 3 shows the relation of the two frailty approaches with survival, independent of motor disability at baseline. Although both approaches remain significantly related with mortality, the frailty phenotype loses much of its predictive value.

## Discussion

In this prospective population-based study, we compared two concepts of frailty in older people with ID: the frailty phenotype and the FI. The FI was more often feasible in the ID population than the frailty phenotype. Both instruments were valid in terms of predicted value for survival; participants classified as frail by either instrument had increased 5-year mortality risks. Even so, people designated as frail by the FI were more likely to decease than those designated as frail by the frailty phenotype. However, the CIs for the FI were wider than the CIs observed for the frailty phenotype, indicating a larger uncertainty in the estimation. Motor disabilities are an important risk factor for mortality. After adjusting the survival models for motor disability, both, but mainly the frailty phenotype, lost predictive value. Previously, we suggested that the FI might be a more suitable concept for this population because of lifelong disabilities (Evenhuis et al. 2013). The current results confirm this suggestion.

The FI could be calculated for 94 % of the participants, whereas the frailty phenotype was feasible in 81 %. For less than a third of the participants (29 %), all frailty phenotype criteria could be measured. This is in agreement with results from studies among assisted-living participants, where nearly 40 % could not complete the assessment (de la Rica-Escuin et al. 2014; Hogan et al. 2012). This dropout was mainly caused by more severe cognitive impairment and chronic comorbidity. This result is in line with results observed in the general population: persons in whom the phenotype cannot be measured completely are significantly more disabled, have more chronic diseases, are more likely to die, and have a higher FI-score (Collerton et al. 2012; Ravindrarajah et al. 2013). On the other hand, dropout for the FI appeared to be random. The agreement between the two instruments was lower compared to other studies (Theou and Rockwood 2015) and the associations between each single frailty phenotype measure and the FI were rather weak, in contrast to others (Hoogendijk et al. 2015).

In accordance with findings in the general population, we found that the predictive value and thereby the criterion validity for the FI is stronger than that of the frailty phenotype (Blodgett et al. 2015; Hogan et al. 2012; Kulminski et al. 2008; Rockwood et al. 2007; Theou and Rockwood 2015; Woo et al. 2012). There are several explanations for our results.

First, the FI has a much broader approach than the frailty phenotype. It includes all factors that are considered important for frailty (e.g., nutritional status, physical activity, energy, cognition) (de Vries et al. 2011; Gobbens et al. 2010). In contrast, the frailty phenotype focuses on physical frailty only. It appears that, among the highly heterogeneous ID population, physical parameters do only explain part of the variance. Indeed, our forward regression analysis implies that, although physical variables are extremely important, disabilities, diseases, and cognition independently add to the explained variance of the model.

Second, and in line with the first suggestion, the frailty phenotype seems to be too determined by mobility limitations. Indeed, in our study the frailty phenotype had only limited additional predictive value to motor disabilities alone. This limits the predictive value of the frailty phenotype, because motor disabilities appear to be less strong predictors for mortality in our ID sample than observed in the general population (Feeny et al. 2012; Majer et al. 2011). Lifelong or early motor impairment, which is common in this population, is likely to be less predictive than motor impairment acquired in later life.

Third, the phenotype approach has the advantage that it focuses on five core clinical features, that are, in theory, easy to measure. Nevertheless, these pre-defined elements are not measurable in all individuals with an ID. This appears less of a problem with the FI approach, which does not require the use of a pre-defined set of variables or even the same number of variables (Rockwood et al. 2006). We were therefore able to design an FI for the ID population, whereas the elements of the frailty phenotype are designed for the general population.

Fourth, we were unable to apply the exact parameters as those proposed in the Cardiovascular Health Study to measure the frailty phenotype. This could have led to an unknown shift in its predictive validity (Theou et al. 2015). In addition, the analyses were applied to participants with at least three elements of the frailty phenotype measured. It is likely that this caused an underestimation of the true frailty prevalence. Measurements that are more feasible for the ID population might have increased the predictive validity of the frailty phenotype. For example, it is known that physical activity is hard to measure with pedometers in people with ID (Hilgenkamp et al. 2012). Using an instrument such as the StepWatch or GPS could have led to more valid results for the element ‘physical activity’ (van Schijndel-Speet et al. submitted).

Nevertheless, overall the frailty phenotype showed a strong relation with mortality, indicating that physical fitness and mobility are important to lengthen the lifespan. Specifically, the mobility-related frailty phenotype items (e.g., walking speed and physical inactivity) were most strongly associated with both mortality and the FI. The group with low physical activity and low walking speed also includes those bound to a wheelchair. These results indicate that even though mobility impairment is less predictive for mortality than observed in the general population, it is a very important risk factor for mortality and overall health (e.g., the FI). It has been shown in the HA-ID study that elements from the frailty phenotype (e.g., grip strength, walking speed) predict disability in mobility and activities of daily living (Oppewal et al. 2014). In the general population, physical activity and fitness can reduce or prevent frailty (Liu and Fielding 2011; Theou et al. 2011). Whether increased physical fitness and activity will also reduce or delay frailty in people with ID needs to be investigated.

The main strength of our study is its large-scale and prospective population-based design, in which we used standardized and internationally accepted methods to measure frailty. Nevertheless, several limitations need to be taken into account. First, although the population was near-representative, older people with ID using specialized support, living independently or with relatives were slightly underrepresented in the HA-ID study. Because of the high correlation between frailty and more severe ID, this underrepresentation might have caused slightly higher prevalence of frailty (Hilgenkamp et al. 2011). Second, we did not take into account time and costs as feasibility aspects. It is very likely that regarding costs, the frailty phenotype is more feasible for clinical practice. Nevertheless, with this study we mainly wanted to better understand frailty and its consequences in this population. For the clinical implementation of any frailty instrument, time and cost should be taken into account. Third, we studied the relation between frailty and survival because mortality is an easily verifiable, dichotomous, and non-arbitrary outcome. Nevertheless, other health outcomes including care need, hospitalization, and disabilities are needed to obtain full insight into the negative consequences of frailty in people with ID. Fourth, the frailty phenotype and the FI are the two most commonly applied concepts. Nevertheless, there are other concepts and frailty instruments that were not included in this study. These measures were chosen because they allow objective measurements, which are needed in a population where only about 25 % is capable of reliable, self-report. In addition, the baseline data of the HA-ID study were already collected before frailty became of interest. Therefore, we were limited to frailty instruments that could be constructed using the available data. In addition, there is value using the two most commonly used measurements in connecting to a large body of published work in order to compare population characteristics. Last, it has been advised to use the FI as a continuous scale, and not apply cut points. Even so, for the sake of comparing the FI with the three frailty strata, proposed by Fried et al., we created three frailty groups applying three different cut points. Nevertheless, the cut points that classified individuals as robust if the FI was below 0.10 or 0.08 resulted in small groups of robust participants and, in line with expectations regarding these robust individuals, limited number of deaths were observed. Using the robust group as a reference group was therefore complicated and resulted in underpowered HRs. We therefore placed most emphasis on the first applied cut value (robust if FI was below 0.20). In order to better understand the agreement and validation of different cut points, a longer follow-up and/or more participants are required.

The two frailty concepts used in our study have a different purpose and different underlying justification. Nevertheless, by comparing the two different concepts, we tried to improve the understanding of frailty in people with ID. The cycle of frailty, which serves as the biological basis of the frailty phenotype, might not be the only relevant aspect in the ID population. Mainly because in this cycle of age-related decline, it is supposed that its individual components are associated with each other and with further physiological losses, disability, dependency, and eventually death. In contrast, in our population motor disabilities can be lifelong and congenital and childhood disabilities are more likely to contribute to frailty than the other way around. For example, it was observed that, according to the frailty phenotype, people with motor disabilities were very likely to also be frail or pre-frail; only 8 % of the participants using a walking aid or wheelchair were classified as robust (Evenhuis et al., 2012). The FI, within clearly defined borders, simply counts how many things are wrong with an individual. Even though the FI also includes lifelong disabilities, it seems that these lifelong problems less influence its validity. Nevertheless, also in people with ID, disabilities increase as a consequence of aging and frailty (Schoufour et al. 2014, 2015). Therefore, identifying frail individuals can assist clinicians in identifying people at risk for adverse health outcomes, who may thereafter benefit from interventions.

Although efforts have been made, there is not yet a validated frailty screening instrument for the ID population (Brehmer-Rinderer et al. 2013). Screening and monitoring the health status of people with ID can potentially have great beneficial effects because recovering from a frail state is complicated, putting more emphasis on early detection and prevention (Rockwood et al. 2011). As the FI provides the highest feasibility and the highest predictive validity, we advise to screen for frailty using an FI like approach. Nevertheless, several steps need to be taken into account before the FI can be applicable to clinical practice. The original FI is composed of 51 items, of which some are not applicable to clinical practice (for example, the block test to measure manual dexterity and the DDS questionnaire to diagnose dysphagia). It should be studied whether the FI remains valid after the removal of less clinically applicable measures. Also, the stability of the FI should be tested by determining the test–retest reliability. Additionally, it is yet unclear if the FI is sensitive to changes over time. In the long run, routinely collected data might be used to calculate an FI and monitor frailty status over time.

With this study, we aimed to better understand frailty in people with ID by applying two different frailty instruments. Our results imply that the used FI is a stronger predictor for mortality than our adaptation of the phenotype in the population of older people with ID. Possible explanations of our findings are that we did not use the exact frailty phenotype variables or that the FI includes multiple health domains. The differences between the two frailty approaches may also be caused by the FI being less determined by lifelong disability and mobility, compared to the frailty phenotype. We suggest that future studies on frailty in people with ID take into account that the feasibility of frailty instruments can be hampered, and adapted instruments are required. Furthermore, lifelong disabilities, such as mobility impairment, could influence the prevalence of frailty and the validity of frailty instruments. Although we acknowledge mobility impairment as a very important aspect of frailty, we suggest using multiple frailty domains, in order to capture the risk for mortality the best. Future research needs to focus on the clinical feasibility of the FI. Particularly, it should be studied whether routinely collected data can be used to construct an FI for people with ID.

## Appendix

See Tables 4, 5, 6, and 7.

**Table 4:** Overview of deficits included in the frailty index (*n* = 982)

#	Deficit	Additional information	Cut-off values and FI scores	Percentage per category	HR (95 % CI)^a^	*p* (for trend)
1	Bladder control	ADL, Completed by professional caregivers of the participants	Incontinent = 1	23.1	3.86 (2.70–5.52)	<0.001
			Sometimes continent = 0.5	24.1	1.39 (0.92–2.08)	
			Continent = 0	52.7	–	
2	Dressing	ADL, Completed by professional caregivers of the participants	Needs help = 1	19.0	5.48 (3.70-8.13)	<0.001
			Partly with help = 0.5	25.8	2.04 (1.38–3.02)	
			No help = 0	55.2	–	
3	Walking stairs	ADL, Completed by professional caregivers of the participants	Needs help = 1	27.6	4.53 (3.12–6.58)	<0.001
			Partly with help = 0.5	20.7	1.75 (1.12–2.73)	
			No help = 0	51.7	–	
4	Bathing	ADL, Completed by professional caregivers of the participants	With help = 1	64.3	2.50 (1.65–3.80)	<0.001
			No help = 0	35.7	–	
5	Transfer bed to chair	ADL, Completed by professional caregivers of the participants	Unable, no sitting balance = 1	9.4	4.79 (3.22–7.12)	<0.001
			Major help = 0.66	3.0	5.17 (3.00–8.90)	
			Minor help = 0.33	13.2	2.71 (1.82–4.01)	
			No help = 0	74.4	–	
6	Groceries	IADL, completed by professional caregivers of the participants	Not independently = 1	51.2	3.08 (1.93–4.93)	<0.001
			With help = 0.5	20.9	1.12 (0.63–1.98)	
			Can do groceries = 0	27.9	–	
7	Housekeeping	IADL, completed by professional caregivers of the participants	Not independently = 1	74.6	3.75 (1.51–9.29)	0.001
			With help = 0.5	15.2	1.58 (0.56–4.51)	
			Can do housekeeping = 0	10.2	–	
8	Falling	Number of falls in the last three months. Information gathered via the professional care giver	>11 falls = 1	1.3	2.61 (1.07–6.41)	0.016
			6–10 falls = 0.75	1.0	1.60 (0.39–6.56)	
			3–5 falls = 0.5	3.8	2.27 (1.27–4.06)	
			1–2 falls = 0.25	17.4	1.31 (0.91–1.87)	
			0 falls = 0	76.4	–	
9	Present at the care center (max 10 shifts per week)	Information gathered via the professional care giver	<3 visits a week = 1	15.7	1.82 (1.27–2.59)	0.001
			≥3 visits a week = 0	84.3	–	
10	Fatigued	ADESS (Dutch translation of the Anxiety, Depression And Mood Scale) over the past six months. Completed by professional caregivers	Very often = 1	6.0	3.36 (3.36–2.06)	<0.001
			Often = 0.66	17.3	1.85 (1.85–1.24)	
			Sometimes = 0.33	30.9	1.67 (1.67–1.17)	
			Never = 0	45.8	–	
11	Listless	ADESS (Dutch translation of the Anxiety, Depression And Mood Scale) over the past six months. Completed by professional caregivers	Very often = 1	3.2	3.54 (2.05–6.13)	<0.001
			Often = 0.66	8.6	1.87 (1.19–2.95)	
			Sometimes = 0.33	23.7	1.46 (1.04–2.04)	
			Never = 0	64.5	–	
12	Panic attacks	ADESS (Dutch translation of the Anxiety, Depression And Mood Scale) over the past six months. Completed by professional caregivers	Very often = 1	3.4	3.47 (2.00–6.06)	<0.001
			Often = 0.66	6.8	1.44 (0.85–2.43)	
			Sometimes = 0.33	13.7	1.71 (1.17–2.50)	
			Never = 0	76.1	–	
13	Decreased food intake, due to loss of appetite, digestive problems, chewing of swallowing difficulties	Mini Nutritional Assessment (MNA) over the past three months. Completed by professional caregivers	Severe decrease in food intake = 1	4.3	2.61 (1.61–4.22)	<0.001
			Moderate decrease in food intake = 0.5	9.5	1.94 (1.31–2.87)	
			No decrease in food intake = 0	86.2	–	
14	Weight loss	Mini Nutritional Assessment (MNA) over the past three months. Completed by professional caregivers	Weight loss greater than 3 kg = 1 Does not know = 0.5	4.7	–	0.023
			Weight loss 1–3 kg = 0.5	24.2	2.05 (1.20–3.52)	
			No weight loss = 0	71.0	1.24 (0.90–1.73)	
15	Fluid intake per day (water, juice, coffee, tea, milk)	Mini Nutritional Assessment (MNA) over the past three months. Completed by professional caregivers	Less than 3 cups = 1	0.5	10.9 (3.94–30.0)	<0.001
			1 to 5 cups = 0.5	14.8	1.13 (0.77–1.65)	
			>5 cups = 0	84.6	–	
16	Calf circumference (CC) in cm	Mini Nutritional Assessment (MNA) Completed by professional caregivers	CC < 31 = 1	21.3	1.92 (1.39–2.67)	<0.001
			CC ≥ 31 = 0	78.7	–	
17	Only eats selected types of food (e.g., pudding, rice)	Screening Tool of Eating Problems (STEP) over the last month. Completed by professional caregivers	> 10 times = 1	4.5	1.73 (1.04–2.90)	<0.001
			Between 1–10 times = 0.5	3.9	2.84 (1.68–4.79)	
			Not at all/not a problem = 0	91.6	–	
18	Only eats small amounts of the presented food	Screening Tool of Eating Problems (STEP) over the last month. Completed by professional caregivers	> 10 times = 1	4.2	1.95 (1.12–3.41)	0.025
			Between 1–10 times = 0.5	12.2	1.39 (0.95–2.04)	
			Not at all/not a problem = 0	83.6	–	
19	Only eats foods of certain textures	Screening Tool of Eating Problems (STEP) over the last month. Completed by professional caregivers	> 10 times = 1	5.4	2.61 (1.65–4.14)	<0.001
			Between 1–10 times = 0.5	2.5	2.36 (1.26–4.39)	
			Not at all/not a problem = 0	92.1	–	
20	Mobility	Provided by professional caregivers	Wheelchair = 1	10.9	4.10 (2.83–5.96)	<0.001
			Walks with support = 0.5	15.3	2.04 (1.40–2.97)	
			Walks independently = 0	73.8	–	
21	CVA	Medical file, last 24 months	Yes = 1	94.0	1.57 (0.95–2.59)	0.080
			No = 0	6.0	–	
22	Coronary heart diseases/heart failure/cardiac dysrhythmia/pacemaker	Medical file, last 24 months	Yes = 1	9.2	2.26 (1.51–3.38)	<0.001
			No = 0	90.8	–	
23	Cancer	Medical file, entire life	Yes = 1	4.9	1.27 (0.70–2.31)	0.43
			No = 0	95.1	–	
24	Asthma/COPD	Medical file, last 24 months, mediation	Yes = 1	13.2	2.27 (1.60–3.24)	<0.001
			No = 0	86.8	–	
25	GERD	Medical file, last 24 months	Yes = 1	20.0	1.52 (1.07–2.15)	0.02
			No = 0	80.0	–	
26	Obstipation	Medical file, last 24 months, medication	Yes = 1	39.7	2.02 (1.48–2.76)	<0.001
			No = 0	60.3	–	
27	Risk for Diabetes Mellitus (DM) or known DM	Medical file, blood glucose levels, medication	DM according to medical file or taking drugs for DM and/or serum glucose ≥ 7 mmol/l = 1	12.4	1.17 (0.76–1.80)	0.75
			No DM according to medial file, no DM drugs and blood glucose 6.1–6.9 = 0.5	2.7	0.90 (0.33–2.43)	
			No DM according to medial file, no DM drugs and blood glucose < 6.1 = 0	84.8	–	
28	Scoliosis	Medical file	Yes = 1	10.6	1.27 (0.81–1.99)	0.30
			No = 0	89.4	–	
29	Visual/Hearing impairments (V/H impairment)	Medical file	At least one severe V/H impairment = 1	24.6	1.76 (1.16–2.67)	0.023
			Two moderate V/H impairment = 1			
			One moderate V/H impairment = 0.5	29.9	1.56 (1.04–2.33)	
			No V/H impairment = 0	45.5	–	
30	Medication use (polypharmacy)	Medical file	≥ 7 drugs = 1	19.8	3.12 (2.13–4.67)	<0.001
			4–6 drugs = 0.5	31.2	1.75 (1.21–2.52)	
			0–3 drugs = 0	48.9	–	
31	Over or under weight	Medical examination	BMI < 18.5 OR > 30 = 1	27.7	1.44 (0.93–2.23)	0.15
			BMI 18.5–20 OR 25–30 = 0.5	41.0	1.01 (0.68–1.51)	
			BMI 20–25 = 0	31.3	–	
32	High blood pressure	Medical file	Yes = 1	21.5	0.94 (0.64–1.39)	0.76
			No = 0	78.5	–	
33	Peripheral atherosclerosis	Medical examination	Ankle Arm index			0.005
			>0.9 = 1	9.9	1.90 (1.15–3.15)	
			0.8–0.9 = 0.5	12.2	1.86 (1.17–2.97)	
			<0.8 = 0	78.0	–	
34	Osteoporosis (*t*-score)	Medical examination	<2.5 = 1	32.7	1.14 (0.72–1.81)	0.109
			−1 till −2.5 = 0.5	38.8	0.72 (0.45–1.14)	
			>−1 = 0	28.6	–	
35	Manual Dexterity (BBT)	Fitness assessment The participants were asked to move as many colored blocks as possible in one minute. The blocks were 2.5 cm^3^ and needed to be moved from one side of a wooden box to the other side	Lowest quartile = 1	26.8	2.75 (1.51–5.00)	<0.001
			Second quartile = 0.66	21.6	1.04 (0.55–1.97)	
			Third quartile = 0.33	26.1	0.83 (0.45–1.53)	
			Highest quartile = 0	25.7	–	
36	Walking speed	Fitness assessment Comfortable walking speed was measured by the average of three records of the time needed to complete 5 meters after 3 meters for acceleration	Slow walking speed was Stratified for height and gender	64.7	3.27 (2.27–4.71)	<0.001
			Male height ≤ 173 cm ≥ 7 s = 1			
			Male height > 173 cm ≥ 6 s = 1			
			Females height ≤ 159 cm ≥ 7 s = 1 Females > 159 cm ≥ 6 s = 1			
			Faster = 0	35.3	–	
			Participant who were not able to succeed the walking speed assessment due to physical limitations were scored positive (score 1) as well			
			Slow walking speed was Stratified for height and gender			
37	Grip strength	Fitness assessment Measured with a Jamar Hand Dynamometer (#5030J1, Sammons Preston Rolyan, USA)	Grip strength was stratified for gender and BMI	52.9 47.1	1.32 (0.86–2.03)	0.21
			Below cut-off values = 1			
			*Male*			
			BMI ≤24: ≥29 kg = 0			
			BMI 24.1–26: ≥30 kg = 0			
			BMI 26.1–28: ≥30 kg = 0			
			BMI >28: ≥32 kg = 0			
			*Female*			
			BMI ≤23: ≥17 kg = 0			
			BMI 23.1–26: ≥17.3 kg = 0			
			BMI 26.1–29: ≥18 kg = 0			
			BMI >29: ≥21 kg = 0			
			Participant who were not able to succeed the grip strength assessment due to physical limitations were scored positive (score 1) as well			
38	Hypercholesterolemia	Medical registry	Yes = 1	10.0	0.58 (0.30–1.11)	0.58
			No = 0	90.0	–	
39	HDL	Blood examination	HDL was stratified for gender	24.6	1.17 (0.69–1.98)	0.49
			*Male*	59.0	0.92 (0.56–1.50)	
			0–0.9 mmol/l = 1	16.4	–	
			0.9–1.55 mmol/L = 0.5			
			>1.55 mmol/L = 0			
			*Female*			
			0–1.1 mmol/l = 1			
			1.1–1.55 mmol/L = 0.5			
			>1.55 mmol/L = 0			
40	Hemoglobin	Blood examination	Stratified for gender	23.8	2.67 (1.87–3.08)	<0.001
			*Male*	76.2	–	
			8.6–10.5 mmol/L = 0			
			<8.6 OR > 10.5 mmol/L = 1			
			*Female*			
			7.5–9.5 mmol/L = 0			
			<7.5 OR > 9.5 mmol/L = 1			
41	Dysphagia	Diagnosis via DDS questionnaire	Severe dysphagia = 1	52.0	2.10 (1.29–3.42)	0.006
			Moderate dysphagia = 0.5	26.1	1.52 (0.88–2.63)	
			No Dysphagia = 0	21.8	–	
42	Hospitalization	Asked in informed consent form. Hospitalization is the past 12 months	>2 = 1	0.6	8.57 (2.59–28.3)	<0.001
			1–2 = 0.5	11.0	1.74 (1.16–2.62)	
			No = 0	88.4	–	
43	Makes a sad/depressing impression	SDZ, completed by professional caregivers Last three months	Often = 1	4.6	4.54 (2.48–7.94)	<0.001
			Several times = 0.66	12.9	1.97 (1.23–3.15)	
			Sometimes = 0.33	36.9	1.87 (1.31–2.68)	
			Never/very rare = 0	45.6	–	
44	Has fun and interest in daily activities	SDZ, completed by professional caregivers Last three months	Never/very rare = 1	4.7	3.06 (1.68–5.57)	0.001
			Sometimes = 0.66	19.2	1.99 (1.30–3.06)	
			Several times = 0.33	39.1	1.40 (0.94–2.08)	
			Often = 0	36.9	–	
45	Sleeps more than regularly (trouble getting out of bed, falls asleep during the day)	SDZ, completed by professional caregivers Last three months	Often = 1	4.5	4.55 (2.68–7.72)	<0.001
			Several times = 0.66	9.8	2.85 (1.84–4.42)	
			Sometimes = 0.33	22.8	1.96 (1.35–2.85)	
			Never/very rare = 0	62.9	–	
46	Fast fatigued/listless	SDZ, completed by professional caregivers Last three months	Often = 1	6.4	4.57 (2.82–7.39)	<0.001
			Several times = 0.66	13.5	2.03 (1.28–3.21)	
			Sometimes = 0.33	35.3	1.62 (1.11–2.37)	
			Never/very rare = 0	44.8	–	
47	Is slow or passive in his/her movements	SDZ, completed by professional caregivers Last three months	Never/very rare = 0 Sometimes = 0.33 Several times = 0.66 Often = 1	6.5 12.1 26.5 54.9	4.66 (2.89–7.50) 2.73 (1.78–4.20) 1.64 (1.11–2.42)	<0.001
48	Knowing which year it is	The Dementia Questionnaire for Mentally Retarded Persons (DMR)	Normally No = 1	57.4	1.55 (1.04–2.27)	0.09
			Sometimes = 0.5	5.3	1.18 (0.57–2.45)	
			Normally Yes = 0	37.2	–	
49	Knowing the way to familiar places	The Dementia Questionnaire for Mentally Retarded Persons (DMR)	Normally No = 1	12.6	3.43 (2.36–5.00)	<0.001
			Sometimes = 0.5	7.6	2.44 (1.56–3.97)	
			Normally Yes = 0	79.8	–	
50	Is seeing group mates	The Dementia Questionnaire for Mentally Retarded Persons (DMR)	Normally No = 1	36.2	2.50 (1.59–3.91)	<0.001
			Sometimes = 0.5	36.5	1.59 (1.03–2.46)	
			Normally Yes = 0	27.3	–	
51	Knowing that today is a weekend or a week day	The Dementia Questionnaire for Mentally Retarded Persons (DMR)	Normally No = 1	23.5	3.56 (2.46–5.16)	<0.001
			Sometimes = 0.5	9.7	2.06 (1.28–3.30)	
			Normally Yes = 0	66.8	–	

^a^ *HR* Hazard ratio, calculated for each provided category using the ‘healthiest’ option as a reference group; HR are adjusted for age, gender, level of ID, and the presence of Down syndrome

**Table 5:** Frailty phenotype variables as originally intended by Fried et al. (2001, 2012) and the adapted frailty phenotype by Evenhuis et al. (2012)

	Original measurement	Applied to the HA-ID study
Weakness	Grip strength: lowest 20 % (by gender, body mass index)	As originally suggested using the Jamar Hand Dynamometer [#5030J1, Sammons Preston Rolyan, Dolgeville, NY]
Shrinking: Weight loss	>10 lbs (4.54 kg) lost unintentionally in prior year	An item of the Mini Nutritional Assessment, weight loss during the past 3 months was assessed on a 4-point rating scale. Losses >3 kg were scored
Exhaustion	Exhaustion by self-report	Exhaustion was estimated using the item “Lacks energy” of the Anxiety, Depression and Mood Scale, using a 4-point rating scale. No exhaustion was classified as no problems or mild problems and exhaustion was classified as moderate problem and severe problem. Because self-report is difficult for a large part of the intellectual disabled population, proxy-based answers were used
Slowness	Walking time/15 ft: slowest 20 % (by gender, height)	As originally suggested. In addition, all participants in a wheelchair and all participants who could not engage in the walking speed assessment because of physical limitations were classified as ‘slow’
Low activity	kcal/week: lowest 20 % males: <383 kcal/week females: <270 kcal/week	All participants walking fewer than 5000 steps/day (sedentary lifestyle) were scored as ‘low activity,’ as were all participants in a wheelchair and all participants who could not engage in the walking speed assessment because of physical limitations

**Table 6:** Hazard ratio’s for 5-year all-cause mortality according to the three level frailty index and frailty phenotype for all participants

Frailty measure	Status	*n* cat	† cat	Single frailty instrument			Both frailty instruments			Motor disability		
				HR (95 % CI)	Wald	*p*	HR (95 % CI)	Wald	*p*	HR (95 % CI)	Wald	*p*
Frailty phenotype	Non-frail	227	20	Reference		Trend: <0.001	Reference		Trend: 0.90	Reference		Trend: 0.04
	Pre-frail	492	100	1.96 (1.10–3.21)	7.21	0.007	1.12 (0.65–1.92)	0.17	0.68	1.62 (0.98–2.68)	3.52	0.06
	Frail	109	45	4.09 (2.35–7.13)	24.8	<0.001	1.16 (0.62–2.19)	0.22	0.64	2.23 (1.20–4.12)	6.48	0.011
Frailty Index	<0.2	320	19	Reference		Trend: <0.001	Reference		<0.001	Reference		<0.001
	0.2–0.35	382	52	2.48 (1.45–4.24)	11.0	<0.001	2.19 (1.24–3.87)	7.20	0.007	2.40 (1.40–4.12)	10.2	<0.001
	>0.35	259	122	11.0 (6.49–18.9)	78.3	<0.001	9.66 (5.23–17.8)	52.5	<0.001	9.13 (5.18–16.1)	58.5	<0.001
Frailty phenotype	Non-frail	227	20	Reference		Trend: <0.001	Reference		Trend: 0.019	Reference		Trend: 0.04
	Pre-frail	492	100	1.96 (1.10–3.21)	7.21	0.007	1.42 (0.84–2.38)	1.71	0.008	1.62 (0.98–2.68)	3.52	0.06
	Frail	109	45	4.09 (2.35–7.13)	24.8	<0.001	2.21 (1.23–4.00)	6.93	0.02	2.23 (1.20–4.12)	6.48	0.011
Frailty Index	≤0.08	33	2	Reference		Trend: <0.001	Reference		Trend: <0.001	Reference		Trend: <0.001
	0.08–0.25	437	33	1.09 (0.26–4.60)	0.02	0.90	0.84 (0.20–3.58)	0.06	0.81	1.09 (0.26–4.59)	0.01	0.91
	≥0.25	491	158	5.07 (1.22–21.0)	5.01	0.025	2.93 (0.67–12.7)	2.06	0.15	4.02 (0.96–16.8)	3.64	0.06
Frailty phenotype	Non-frail	227	20	Reference		Trend: <0.001	Reference		Trend: 0.001	Reference		Trend: 0.04
	Pre-frail	492	100	1.96 (1.10–3.21)	7.21	0.007	1.46 (0.87–2.43)	2.05	0.54	1.62 (0.98–2.68)	3.52	0.06
	Frail	109	45	4.09 (2.35–7.13)	24.8	<0.001	2.56 (1.44–4.56)	10.2	0.03	2.23 (1.20–4.12)	6.48	0.011
Frailty index	≤0.10	64	2	Reference		Trend: <0.001	Reference		Trend: <0.001	Reference		Trend: <0.001
	0.10–0.21	281	19	1.94 (0.45–8.38)	0.79	0.37	1.59 (0.36–6.96)	0.39	0.15	1.97 (0.46–8.51)	0.83	0.36
	≥0.21	616	172	7.80 (1.90–32.0)	8.12	0.004	4.79 (1.12–20.4)	4.48	0.001	6.28 (1.52–26.0)	6.44	0.01

*Note* *HR* hazard ratio, *CI* confidence interval, *†* the total number of deceased participants, the non-frail state was used a reference category for each frailty instrument. All models were adjusted for age, gender, level of ID, and Down syndrome. The model *motor disability* was adjusted for the level of motor impairment, ‘no walking impairment’ was used as a reference category; the model for motor disability included only the frailty phenotype or the frailty index. For the analysis with both frailty instruments, 818 participants were included. For the Motor disability analysis, 7 participants were excluded because they had no data on motor disabilities

**Table 7:** Forward Cox proportional Hazard model using all available frailty variables as independent predictors for survival

Frailty item	Wald	*p* value	HR	Lower CI	Upper CI
Walking stairs	20.2	<0.001	2.39	1.63	3.49
Present at day care center	22.0	<0.001	2.17	1.57	3.00
Panic attacks	4.30	<0.001	1.66	1.03	2.67
Weight loss	9.50	0.002	2.09	1.31	3.33
Cardiovascular	19.8	<0.001	2.27	1.58	3.25
Asthma/COPD	12.2	<0.001	1.86	1.31	2.62
Visual/hearing impairments	8.88	0.003	1.74	1.21	2.50
hemoglobin	16.7	<0.001	1.89	1.39	2.56
Sleeps more than regularly	15.3	<0.001	2.69	1.64	4.42
Slow or passive	15.4	<0.001	2.70	1.64	4.42
Knowing week day or weekend day	19.2	<0.001	2.08	1.50	2.88
Hospitalization	4.96	0.026	2.13	1.10	4.14
Fast fatigue	5.87	0.015	0.47	0.26	0.87
